# Vascular Acrosyndromes Associated With Prolonged Tumor Response in Advanced Lung Cancer Patients During Treatment With Antimetabolites: A Report of Two Cases

**DOI:** 10.3389/fonc.2021.644282

**Published:** 2021-04-01

**Authors:** Margaux Geier, Hélène Babey, Lucie Monceau-Baroux, Gilles Quéré, Renaud Descourt, Divi Cornec, Gilles Robinet

**Affiliations:** ^1^ Department of Oncology, Centre Hospitalier Regional Universitaire (CHRU) Morvan, University Hospital of Brest, Brest, France; ^2^ Department of Rheumatology, CHRU Cavale Blanche, University Hospital of Brest, Brest, France

**Keywords:** digital ischemia, antimetabolite agents, non-small cell lung cancer, case report, cancer response

## Abstract

**Background:**

Pemetrexed and gemcitabine are both antimetabolites drugs approved in advanced non-small cell lung cancer (NSCLC). Their toxicity profile is well known. However, rare vascular side effects can occur such as vascular acrosyndromes and especially digital ischemia. The cause of this disfiguring and painful event is still controversial. Amputation is frequently required and has been described as a predictor of poor survival outcomes.

**Case Presentation:**

This report presents two cases of vascular acrosyndrome in NSCLC patients during treatment with antimetabolites (pemetrexed and gemcitabine). Patients presented severe digital ischemia having required prostacyclin analog and chemotherapy discontinuation. In one case, symptoms improved while in the other case symptoms persisted. Both patients experienced prolonged tumor response. These findings suggest a multifactorial mechanism behind digital necrosis including an autoimmune process, which could lead to prolonged tumor control as described with immune checkpoint inhibitors.

**Conclusion:**

Severe vascular acrosyndrome such as digital ischemia can occur in lung cancer patients treated with antimetabolites. Awareness needs to be raised when using these drugs in patients with predisposing factors. Whether occurrence of chemotherapy-induced immune vascular side effects might explain prolonged tumor response deserves further investigations.

## Background

Gemcitabine is a nucleosid analog that inhibits DNA synthesis and repair. Pemetrexed is a new generation multifolate antagonist that directly affects the enzymes involved in cell activation and cell division. Both antimetabolite drugs have displayed significant antitumor activity in metastatic non-small cell lung cancer (NSCLC) when combined with platinum-based chemotherapy (1, 2). They are also approved as single-agent with mild manageable toxicity. Side effects commonly observed with gemcitabine are myelosuppression, influenza-like syndrome and vascular toxicity. Pemetrexed is primarily responsible of hematological, hepatic and renal toxicities (1, 2). Herein, we report two cases of NSCLC patients treated with these antimetabolite drugs, who experienced severe vascular acrosyndromes but also prolonged tumor response.

## Case Presentation 1

**Figure 1:**
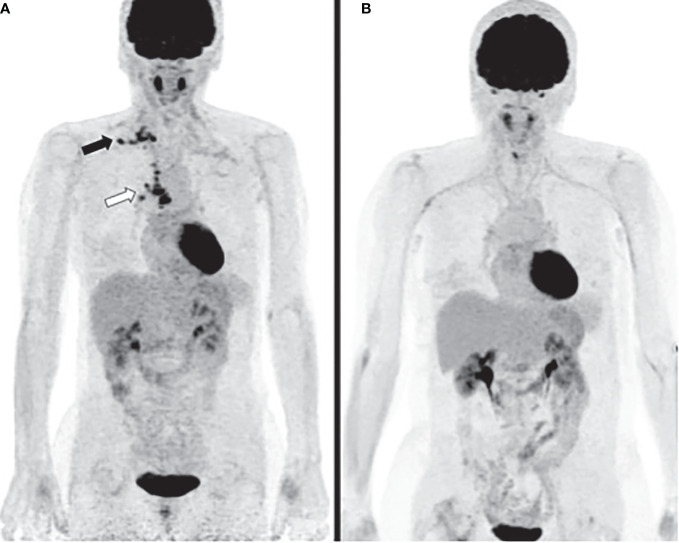
Case 1 **(A)** PET computed tomography (CT) at diagnosis reveals hypermetabolic activities of right hilar, mediastinal (white arrow) and supra-clavicular lymph nodes (black arrow). **(B)** PET CT three years after diagnosis shows complete metabolic response.

A 58-year-old woman presented in November 2016 with a diagnosis of KRAS-mutant advanced lung adenocarcinoma (**Figure 1A**). Her medical comorbidities included hypertension controlled with calcium channel blocker, and active smoking. She reported Raynaud’s like symptoms without etiological orientation that appeared 6 months earlier. The patient was treated with six cycles of cisplatin and pemetrexed, resulting in complete tumor response.

Prior to start maintenance pemetrexed at 500 mg/m^2^ every 3 weeks, she reported recurrence of a vascular acrosyndrome of both hands. She described episodic pain, numbness, tumefaction and cyanosis of all digits and was therefore admitted in Department of Dermatology for evaluation. The patient reported xerostomia as systemic symptoms. General health status was good. Physical examination revealed sclerodactyly of hands, distal ulceration of the fourth right and left digits. Raynaud’s phenomenon was found in the upper and lower limbs without telangiectasia or subcutaneous calcifications. Her chest, heart, abdomen, and lymph node stations examination results were normal.

Laboratory studies showed macrocytic anemia with hemoglobin concentration of 11.8 g/dl. Other routine tests (electrolytes, creatinin, liver function, and hemostasis) were normal. Urinary sediment, CPK, C3-, and C4-complement, cryoglobulins, and ANCA did not show any abnormality. An antinuclear antibody (ANA) test was performed, with a weakly positive (>1/320; N: <1/160) speckled pattern. Anti-extractable nuclear antigen antibodies were negative (especially no anti-Scl70 or anti-centromere antibodies). Anti-phosphatidylserine/prothrombin (aPS/PT) antibodies were mildly positive (21 U; N: <15), but other antiphospholipid antibodies were not detected. The contrast CT scan of thorax showed complete tumor response without interstitial lung disease associated. Lung function test and echocardiogram were normal. Capillaroscopic imaging was normal. Arterial Doppler ultrasound revealed a normal macro-circulation of the upper and lower limbs.

Paraneoplastic syndrome was initially suspected and chemotherapy was pursued. Shortly after, she experienced significant pain exacerbation and developed ulceration of fourth left digit, left heel and toes. Patient received prostacyclin analog iloprost trometamol (20 mg/day for three weeks) with gradually improvement of symptoms. Pemetrexed infusions were stopped since chemotherapy-induced endothelial dysfunction was suspected. Tumor response remained complete more than 3 years after chemotherapy discontinuation and no further episode of digital ischemia occurred (**Figure 1B**).

## Case Presentation 2

**Figure 2:**
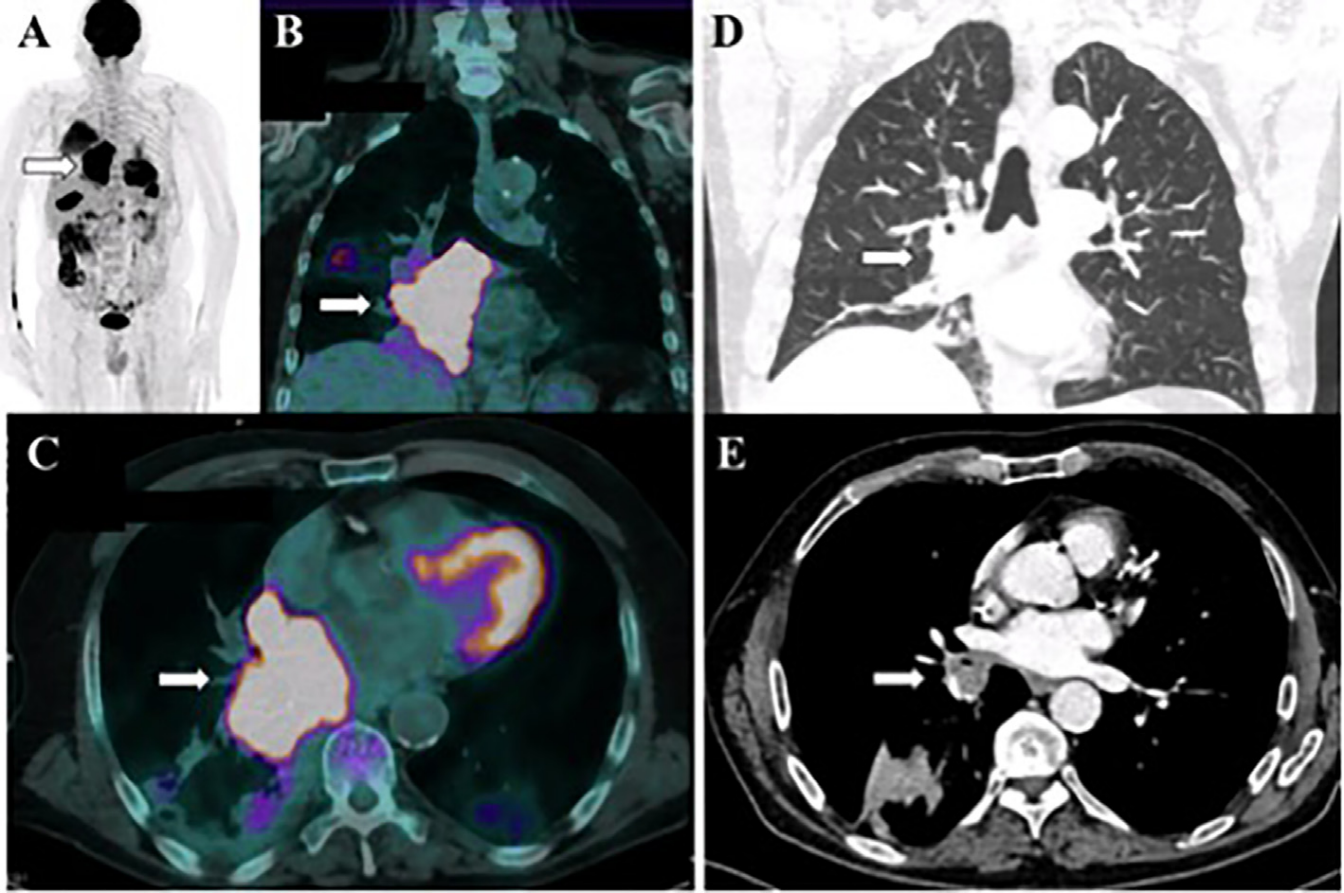
Case 2 **(A-C)** PET CT at diagnosis shows an intensely hypermetabolic mass at right lower lobe adjacent to mediastinum (white arrows). **(D, E)** CT image of the chest performed 16 months after diagnosis shows partial response of the primary lung tumor (white arrows).

A 69-year-old man presented to the emergency department in August 2018 with acute limb ischemia. He was a heavy smoker and had a history of high blood pressure and peripheral arterial occlusive disease (PAOD). On examination, his left foot appeared cold with hypoesthesia along the medial aspect of the forefoot. Arterial and venous Doppler ultrasound of the lower limbs revealed critical ischemia of the left lower limb related to an occlusion of left external iliac artery associated with left proximal deep vein thrombosis. Aortic CT angiography confirmed arterial occlusion with fortuitous diagnosis of a right lung tumor mass. Patient underwent peripheral artery bypass and anticoagulant therapy was initiated resulting in the improvement of his symptoms. A diagnosis of PD-L1 negative metastatic lung squamous cell carcinoma was subsequently made (**Figures 2A–C**) and gemcitabine-platinum combination was started. Partial objective tumor response was observed after four cycles and maintenance gemcitabine was pursued. Between first and second cycle of maintenance, he described occurrence of pain, cyanosis and hypoesthesia of all digits except thumbs with distal ulceration of the second right digit but also numbness and edema of the left big toe.

Laboratory tests did not find any etiology (negative ANCA, absence of cryoglobulins). To note, anti-SSA antibodies were weakly positive in serum (12 U/ml; N: <10). Arterial Doppler ultrasound was normal. Nail fold capillaroscopy was not performed. Based on the hypothesis of gemcitabine-induced vasculitis, patient discontinued chemotherapy. A prolonged infusion of iloprost trometamol was then administered with incomplete improvement of vascular acrosyndrome.

Despite this event, patient had no sign of cancer recurrence and duration of tumor objective response achieved 16 months (**Figures 2D, E** **)**. One year after chemotherapy discontinuation, patient described persistent pain in the left foot. Electrophysiological test was therefore performed and highlighted multiple mononeuropathy. Anti-SSA antibodies were detected in serum (66 U/ml; N: <10). Neurological manifestation of Sjögren’s syndrome was suspected but not confirmed after performance of salivary glands biopsy. Unfortunately, cancer progressed in April 2020 and second-line anti–PD-1 was initiated. After the first infusion of pembrolizumab, patient described occurrence of pain regarding his right foot with intermittent claudication. CT angiography confirmed worsening of PAOD and patient underwent a second peripheral artery bypass resulting in control of symptoms. After 6 cycles of anti–PD-1, progressive disease was observed and chemotherapy with paclitaxel was initiated.

## Discussion

In the present study, we report two rare cases of advanced NSCLC patients presenting vascular acrosyndrome leading to digital ischemia that occurred during first line antimetabolite-based chemotherapy sequence. In the first case, symptoms resolved after discontinuation of pemetrexed, unlike in the second case where symptoms persisted despite gemcitabine discontinuation. Fortunately, no amputation was necessary. In both cases, antimetabolite infusions were stopped and patients experienced prolonged tumor response (not reached after 3 years and 16 months, for case 1 and 2 respectively).

Digital ischemia is an uncommon and painful condition with a negative impact on patient’s quality of life but is rare among cancer patients. Etiologies of vascular acrosyndromes are numerous and include connective disorders, vasculitis, hematological diseases, paraneoplastic syndromes, drugs, vasculopathy, infectious diseases, trauma or embolic diseases; all complicated by secondary vasospasm (3). Here, we discuss the potential multifactorial mechanisms underlying vascular acrosyndrome worsened by antimetabolites administration as antineoplastic agents and the relationship with associated prolonged tumor response.

Cardiovascular risk factors constitute an important cause of vascular acrosyndromes (4). Indeed, NSCLC patients often have smoking history, arterial hypertension, hypercholesterolemia, and/or diabetes with atherosclerotic comorbidities leading to PAOD and ischemic events, as described in our case 2. However, atherosclerosis and cholesterol embolization syndrome are rarely the cause of digital ischemia but more often implicated in toe necrosis (5).

Patient’s ischemic symptoms could also be an adverse consequence of cumulative toxicity of antimetabolites resulting in endothelial dysfunction and hypercoagulability. Indeed, chemotherapy-induced endothelial lesions have been previously reported. Specific chemotherapies such as cyclophosphamide, methotrexate, 5-fluorouracil, gemcitabine, cis/carboplatin, pemetrexed could be involved and have been implicated in thrombosis and thrombembolic events (6). Important vascular side effects have been related to the use of gemcitabine, probably higher than previously estimated and currently largely reported in literature. Among them, venous and arterial thromboembolic events, necrotizing vasculitis, thrombotic microangiopathy and severe systemic capillary leak syndrome have been described (7–10). Vénat-Bouvet et al. reported cases of probable thrombotic microangiopathy and digital necrosis due to gemcitabine perfusion (11). In these cases, chemotherapy was withdrawn, resulting in resolution of symptoms, despite cancer progression. Vascular toxicity of pemetrexed is less frequently reported in literature. Gupta et al. described long-term exposure to pemetrexed in the case of a 65-year-old patient with a diagnosis of metastatic lung adenocarcinoma leading to multiple digital infarctions requiring amputation (12). Authors concluded that Raynaud’s phenomenon (RP) in the initial stages may be the consequence of pemetrexed-induced endothelial damage and that special attention should be given to patients on maintenance pemetrexed who complained about painful digits. In our case 1, occurrence of digital ischemia was probably due to pemetrexed vascular side effect as indicated by resolving microcirculation in all digits after chemotherapy discontinuation and the intervention with vasodilating agent iloprost.

Occurrence of digital ischemia in patients with active malignancy may also be considered as a paraneoplastic disorder, especially in the case of adenocarcinoma, squamous cell carcinoma or hematologic malignancies (13, 14). However, lung carcinoma-related digital necrosis remains an uncommon paraneoplastic feature and is considered more often as a consequence of NSCLC (15, 16). It may be a complication of its own or may be associated with RP. The presence of antinuclear and antiphospholipid antibodies remains uncertain. Multiple mechanisms have been suggested to explain vascular acrosyndrome associated with malignancy (14). The main one proposed is a neoplastic involvement of the cervical sympathetic trunk, with resulting peripheral vasospasm or overproduction of vasoconstrictive substances by tumor cells (13, 17). A thromboembolic mechanism, with either migration of tumor fragments or hyperviscosity, hypercoagulability and spontaneous platelet aggregation have also been discussed (13). In several reports of patients with paraneoplastic vascular acrosyndrome, the vasospastic complications often improve with initiation of specific anti-cancer therapy (18). In our cases and especially in case 1 with pre-existing RP, this etiology was unlikely the cause of patient’s digital manifestations, as patients had objective tumor response at the time of worsening of symptoms.

Hypothesis of an immunologic mechanism is also suggested (13). Indeed, malignant diseases may promote autoimmunity by generation of autoantibodies against various autoantigens, resulting in complement activation in contact with the arterial wall. Furthermore, the detection of ANA or abnormal cryoglobulins might be explained by an altered function of suppressor T cells and by proliferation of monoclonal B lymphoid cells (19, 20). Finally, diversity of the major histocompatibility complex and innate immune gene expression have a significant positive correlation with resistance or susceptibility to various types of cancers (21).

In the present cases, potential pre-existing connective disorder might have influenced the occurrence of digital ischemia. In patient 1, there was a weak evidence of an underlying autoimmune process due to borderline ANA with positive aPS/PT antibodies suggestive of an antiphospholipid antibody syndrome, and a diagnosis of paraneoplastic systemic sclerosis (SSc) could also be discussed with recent-onset RP and sclerodactily, but there was no specific autoantibody and capillaroscopy was normal. Sjögren’s syndrome could be suspected in case 2 with positive anti-SSA antibodies. Patients with pre-existing rheumatic disorders such as SSc are at high risk for developing digital ulcers because of their latent vascular disease with impairment of microcirculation and associated RP (22). The occurrence of multiple acral ischemic lesions in a SSc patient after receiving platinum-based chemotherapy plus gemcitabine for NSCLC has already been described (23). Thereby, several authors recommend caution when administering chemotherapy in subjects with autoimmune disorders. These cases could highlight interaction of chemotherapy toxicity and immune-mediated vascular disease leading to digital ischemia.

The association between treatment toxicity and clinical outcomes has long been a concern in cancer patients. It is however possible that relation between treatment-related toxicities and outcomes varies according to therapeutic modality or specific cancer type. Prolonged tumor response associated with immune-related adverse events (IRAEs) is well described in cancer patients treated with immune checkpoint inhibitors (ICIs) (24). Restoration of antitumor immunity during treatment with ICIs leads to a broad spectrum of manifestations such as hypophysitis, colitis, pneumonitis but also vasculitis of medium and large vessels (24). Conversely, reports of small vessel vasculitis involving the digits are rare. Several recent reports described the development of acral vascular necrosis with ICIs, without preexisting autoimmune disease prior initiation of immunotherapy (25, 26). Khaddour et al. reported the occurrence of digital necrosis in a patient with lung adenocarcinoma treated with pembrolizumab (26). One hypothesis was the mechanism of action of ICIs, potentially leading to disruption of immune tolerance with stimulation of T cell populations or autoantibody formation against various antigens such as endothelial cells, which might induce vasculitis disorders. Moreover, an autoimmune etiology of digital ischemic symptoms during ICIs treatment is supported in literature as suppressive dose of steroids might resolve acral ischemia (27, 28).

Here, we postulate that antimetabolites induced both vascular IRAE and prolonged response, as reported with ICIs. Indeed, preclinical studies have revealed that the mechanism of action of cancer drugs depends on several off-target effects, especially directed to the host immune system. These off-target interactions contribute to cancer cells elimination (29, 30). Gemcitabine has a powerful downregulation effect on the immunosuppressive microenvironment (31, 32). It notably depletes regulatory T-cells and selectively kills myeloid-derived suppressor cells, thereby relieving immunosuppression and enhancing CD8 T-cell-dependent anticancer immune responses (31). The influence of pemetrexed on the immune-mediated antitumor response is however currently underrated. Novosiadly et al. evaluated the preclinical profile of pemetrexed on immune response in tumor microenvironment (33). They concluded that pemetrexed enhanced T cell-mediated immunogenic cell death and increased activation and metabolic fitness of T cells. These effects enhanced anti-tumor efficacy in combination with PD-1 pathway blockade. According to these results, we suggest that chemotherapies could also lead to vascular IRAEs and to a favorable clinical course (7). Nonetheless, the literature data are currently lacking to confirm the robustness of this hypothesis.

To our knowledge, our reports are the first ones to describe development of vascular acrosyndrome with the use of antimetabolites in NSCLC patients experiencing prolonged tumor response despite chemotherapy discontinuation. In the future, special attention should be paid to NSCLC patients in maintenance therapy, especially since FDA approval pemetrexed-pembrolizumab combination as first-line treatment in advanced non-squamous lung carcinoma. Indeed, systematic use of ICIs combined with antimetabolite could theoretically increase occurrence of digital ischemia, as their positive interaction may enhance immunogenic response.

## Conclusion

Vascular acrosyndromes are a rare complication of antimetabolite chemotherapeutic drugs. Awareness needs to be raised when using these anticancer agents. They may rarely promote digital infarction for patients with predisposing factors. Whether occurrence of chemotherapy-induced immune vascular side effects might explain prolonged tumor response deserves further investigations.

## Data Availability Statement

The original contributions presented in the study are included in the article/supplementary material. Further inquiries can be directed to the corresponding author.

## Ethics Statement

Written informed consent was obtained from the individual(s) for the publication of any potentially identifiable images or data included in this article.

## Author Contributions

Category 1: Conception and design of study: MG and GR. Acquisition of data: MG and GR. Analysis and/or interpretation of data: MG, RD, DC and GR. Category 2: Drafting the manuscript: MG and GR. Revising the manuscript critically for important intellectual content: MG, HB, LM-B, GQ, RD, DC and GR. Category 3: Approval of the version of the manuscript to be published : MG, HB, LM-B, GQ, RD, DC and GR.

## Conflict of Interest

The authors declare that the research was conducted in the absence of any commercial or financial relationships that could be construed as a potential conflict of interest.
